# Discordance of global assessment between the patients and physicians predicts 9-year pain-related outcomes in rheumatoid arthritis patients

**DOI:** 10.3389/fmed.2023.1189748

**Published:** 2023-06-19

**Authors:** Kazuhiro Hayashi, Kenji Miki, Kenrin Shi, Masao Yukioka, Takehiro Hirayama, Kohei Tsujimoto, Takao Takeuchi, Yasuhisa Hayaishi, Masahiro Hayaishi

**Affiliations:** ^1^Department of Physical Therapy and Rehabilitation Science, University of Iowa, Iowa City, IA, United States; ^2^Center for Pain Management, Hayaishi Hospital, Osaka, Japan; ^3^Faculty of Health Science, Osaka Yukioka College of Health Science, Ibaraki, Japan; ^4^Japan Pain Foundation, Tokyo, Japan; ^5^Department of Rheumatology, Tenjin Orthopaedics and Rheumatology, Osaka, Japan; ^6^Department of Rheumatology, Yukioka Hospital, Osaka, Japan; ^7^Department of Respiratory Medicine and Clinical Immunology, Graduate School of Medicine, Osaka University, Suita, Japan; ^8^Department of Rheumatology, Hayaishi Hospital, Osaka, Japan

**Keywords:** arthritis rheumatoid, communication, physicians, prognosis, quality of life

## Abstract

**Introduction:**

Perspectives regarding the disease state often differ between patients with rheumatoid arthritis (RA) and physicians. The aim of the present longitudinal cohort study was to investigate the impact of the discordance in global assessments between patients and physicians on 9-year pain-related outcomes in patients with rheumatoid arthritis.

**Method:**

Sixty-eight consecutive outpatients with rheumatoid arthritis on their first visit to a tertiary center were included. Baseline measurements included demographic data, drugs used, disease activity, and a modified Health Assessment Questionnaire (mHAQ). Discordance in global assessment between patients and physicians at baseline was defined as 10 mm higher in the patient global assessment (PGA) than in the physician global assessment. A 9-year follow-up assessment included pain intensity, the European Quality of Life 5 Dimensions 3 Level (EQ-5D-3L) scale, Pain Catastrophizing Scale (PCS), Hospital Anxiety and Depression Scale (HADS), Pain Disability Assessment Scale (PDAS), and Pain Self-Efficacy Questionnaire (PSEQ).

**Results:**

The number of patients with discordance was 26 (38%) in 68 patients. Patients with a 10 mm higher PGA than the physician global assessment at baseline measurements had significantly worse pain intensity, PCS score, PSEQ score, and EQ-5D-3L score measurements at the 9-year follow-up than those with concordance. A higher mHAQ score and 10 mm higher PGA at baseline were significantly independently associated with the EQ-5D-3L scale score and pain intensity at the 9-year follow-up.

**Conclusion:**

This longitudinal cohort study suggested that discordance in global assessment between patients and physicians modestly predicted worse 9-year pain-related outcomes in patients with rheumatoid arthritis.

## 1. Introduction

Rheumatoid arthritis (RA) is classified based on joint distribution, serology, symptom duration, and acute-phase reactants according to the 2010 American College of Rheumatology/European League Against Rheumatism classification criteria (1). RA has an incidence of 0.5–1%, affects women two to three times more often than men, and occurs at any age (2, 3). RA involves chronic inflammation of the synovial membrane, with attendant worsening in physical function, cumulative comorbid risk, ability to work, and quality of life (2–7). The experience of pain in RA is multifactorial, and it can be due to structural changes in the joint as well as pain-related psychological factors (8). Treatment options include education complemented by physical activity and exercise, psychological and social interventions, sleep hygiene education, weight management, orthotics, pharmacological and joint-specific treatment options such as a local injection, and interdisciplinary treatment (9). The prognosis factors in RA are gender, disease activity, psychological factors, and education level (10–12). Routine care for RA includes a comprehensive assessment of specific symptoms (13, 14).

The global assessment of disease by patients and physicians constitutes a part of the disease activity measures for RA (1). Interestingly, the perspective regarding disease state often differs between patients and physicians (15, 16). The frequency of discordance in global assessments between patients and physicians is 36–51% (17). The cutoff defining discordance is inconsistent among countries and studies, ranging from 5 mm to 30 mm on a 0–100 mm visual analog scale (17). Forty-five percent of Asian patients with RA showed a discordance of 10 mm higher in patients than physicians (18). The discordance is influenced by the tender joint count, swollen joint count, pain, fatigue, health literacy, and depressive symptoms (15–18). One longitudinal study showed that discordance before treatment was significantly associated with pain, disease activity, and activity of daily living after treatment (18). This suggests the importance of discordance in treatment outcomes in patients with RA, although the association was based on a univariable test over 12 months. The impact of discordance on treatment outcomes should be considered along with confounding factors, including age and disease activity. However, no studies have evaluated the association between the discordance in global assessment between patients and physicians and long-term treatment outcomes in patients with RA.

This longitudinal cohort study aimed to investigate the hypothesis that discordance in global assessments between patients and physicians predicts worse 9-year pain-related outcomes in patients with RA.

## 2. Materials and methods

### 2.1. Study design

Baseline measurements were assessed face-to-face during the first visit to a tertiary center by a doctor. Follow-up measurements were assessed by mail survey 9 years after the first visit.

The sample size was calculated using the G*Power software (version 3.1.9.2; Franz Faul, Kiel University, Kiel, Germany). The minimum number of subjects was estimated to be 68 for an α-level of 0.05, and a power (1–β) of 0.80 (18).

All methods of the present longitudinal cohort study were performed following the Strengthening the Reporting of Observational Studies in Epidemiology (STROBE) guidelines (19). This study was approved by the Research Ethics Committee of Hayaishi Hospital and all the patients provided written informed consent for this study.

### 2.2. Participants

Participants were purposively and consecutively recruited during their doctor visits at our tertiary center between November 2012 and February 2013. Inclusion criteria were as follows: (1) older than 20 years of age, (2) first visit to our tertiary center, and (3) a diagnosis of established RA, more than 1 year of disease duration, by a medical doctor, based on the American College of Rheumatology/European League Against Rheumatism classification criteria (1).

Exclusion criteria were as follows: (1) cancer-related pain, neurological disease, and evidence of bone fractures; (2) recent surgery within the past 6 months; (3) consuming medication associated with dementia; (4) poor Japanese language comprehension; and (5) not returning or not completing the follow-up measurement by mail. All inclusion and exclusion criteria were assessed by the referring physicians.

### 2.3. Treatment

All patients received the usual treatment following recommendations from the clinical practice guidelines (20). Treatment in the clinic was performed at least once every 3 months by orthopedics and physical therapists. The treatment included advice to remain active with education and reassurance as first-line care. If patients needed second-line care, non-pharmacological treatment was attempted before pharmacological treatment. Pharmacological treatments were administered at the lowest effective dose for the shortest period possible.

### 2.4. Baseline measurement

All baseline measurements were collected during the first visit to the tertiary center. Demographic data included age, sex, body mass index, disease duration of RA, Steinbrocker-class classification (21), and drug use (non-steroidal anti-inflammatory drugs, glucocorticoids, methotrexate, and biological agents).

Disease activity was assessed using the Disease Activity Score 28 joint count, erythrocyte sedimentation rate (DAS28-ESR), and Simplified Disease Activity Index (22). Calculation of the DAS28-ESR and Simplified Disease Activity Index was used in the outcome parameters: tender joint count and swollen joint count based on a 28-joint assessment, patient global assessment of disease activity (PGA) with a visual analog scale of 0–100 mm, physicians global assessment of disease activity with a visual analog scale of 0–100 mm, C-reactive protein, and ESR. The questions of global assessment of disease activity are “How do you estimate your disease activity?” The discordance of the global assessment between patients and physicians was defined as a 10 mm higher PGA than in the physician global assessment (17, 18).

Patient satisfaction regarding the activities of daily living was assessed using a modified Health Assessment Questionnaire (mHAQ) (23). The mHAQ score was calculated as the mean of the scores for eight activities of daily living.

### 2.5. Follow-up measurement

All follow-up measurements were performed by mail 9 years after the first visit. The sender and return addresses were tertiary centers. The patients were instructed by a letter included in the questionnaire package.

Quality of life and pain intensity were the primary outcome measures. Quality of life was measured using the European Quality of Life 5 Dimensions 3 Level (EQ-5D-3L) scale, a generic scale used worldwide that assesses health in five dimensions: mobility, self-care, usual activities, pain/discomfort, and anxiety/depression (13, 14). Each domain was assessed using a single question with three possible responses: no problems, problems, and serious health problems. The combination of all the possible dimensions and levels resulted in 243 unique health states. It can be converted into EQ-5D-3L scale scores ranging from −0.111 to 1.00. A score of 0 represents death, and 1.00 represents a state of full health. Pain intensity was measured using a 0–10 pain numerical rating scale (NRS) (24). The scale, which ranged from 0 to 10, was used as an indicator of the average level of pain during the day. The scale was labeled at the anchor points, with 0 indicating “no pain” and 10 indicating “worst possible pain.”

Secondary outcomes were measured using the Japanese version of the following questionnaires: Pain Catastrophizing Scale (PCS) (25, 26), Hospital Anxiety and Depression Scale (HADS) (27, 28), Pain Disability Assessment Scale (PDAS) (29), Pain Self-Efficacy Questionnaire (PSEQ) (30, 31), and working status.

The PCS consists of 13 items (25, 26). The participants rated how frequently they experienced emotions such as rumination (e.g., “I keep thinking about how much it hurts”), magnification (e.g., “I wonder whether something serious may happen”), and helplessness (e.g., “There is nothing I can do to reduce the intensity of the pain”). The total PCS score ranged from 0 to 52, with higher scores indicating higher levels of catastrophizing.

The HADS was designed to assess two separate dimensions: anxiety and depression (27, 28). The HADS consists of 14 items, and the anxiety and depression subscales each include seven items. A four-point response scale (from 0 representing the absence of symptoms to 3 representing maximum symptoms) was used, with possible scores for each subscale ranging from 0 to 21.

The PDAS assesses the degree to which chronic pain interferes with various daily activities during the past week (29). The PDAS includes 20 items reflecting pain interference in a broad range of daily activities, and respondents indicate the extent to which pain interferes. Scores on the total PDAS ranged from 0 to 60, with higher scores indicating higher levels of pain interference.

The PSEQ scores were designed to assess the degree of confidence in performing several activities despite pain (30, 31). The PSEQ consisted of 10 items. The total PSEQ score ranges from 0 to 60, with lower scores indicating lower levels of self-efficacy for functioning despite the pain.

### 2.6. Statistical analysis

All continuous data are expressed as means and standard deviations. The normality of the distribution was evaluated using the Shapiro-Wilk test for the continuous variables. Univariate and multivariate tests were used for comparisons. The categorical variables included dummy variables. The correlation between variables was analyzed using Pearson’s correlation coefficient test. Multivariate analysis was used to investigate variables with *p* < 0.1 in the univariable analysis. Four variables were analyzed in the multivariable analysis for the EQ-5D-3L scale score: mHAQ, 10 mm PGA higher, biological agents, and Simplified Disease Activity Index scores. Three variables were analyzed in the multivariate analysis for Pain-NRS value: mHAQ, 10 mm PGA higher, and biological agents. The multicollinearity of the variables was also assessed (correlation coefficient < 0.9).

Data were analyzed using SPSS (version 27.0 for Microsoft Windows; SPSS, Chicago, IL, USA). A *p*-value of < 0.05 was considered to be statistically significant.

## 3. Result

**FIGURE 1:**
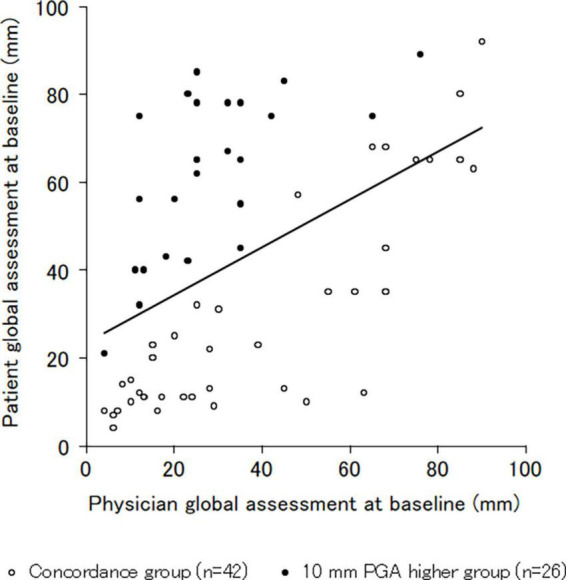
Correlation of the patient and physician global assessment. The physician global assessment value was significantly correlated with the mean patient global assessment value, with a moderate correlation coefficient (*r* = 0.506, *p* < 0.001). *Significance level was set at <5%.

The survey response rate was 63% (*n* = 68 of 107). The patient characteristics are shown in Table 1. The mean patient age was 62 years. Of the 68 patients, 62 (91%) were women. The mean PGA value was 42.55 mm, while the mean physician global assessment value was 35.13 mm (7.42 mm higher in the patient than the physician). The physician’s global assessment value was significantly correlated with the mean PGA value with a moderate correlation coefficient (*r* = 0.506, *p* < 0.001*) (Figure 1). The number of patients with 10 mm higher PGA than the physician’s global assessment was 26 of 68 (38%).

**TABLE 1:** Comparison of data between patients with concordance and those with 10 mm PGA higher than physician global assessment.

	Overall (*n* = 68)	Concordance (*n* = 42)	10 mm PGA higher (*n* = 26)	Odds ratio (95% CI)	*p*-value
Baseline					
Age (years)	62.69 (10.46)	62.57 (10.09)	62.88 (11.24)	0.313 [−4.940 to 5.566]	0.906
≤49 [n (%)]	9 (13%)	5 (11%)	4 (15%)	0.035 [−0.136 to 0.206]	0.686
50–59 [n (%)]	14 (20%)	8 (19%)	6 (23%)	0.040 [−0.164 to 0.245]	0.695
60–69 [n (%)]	28 (41%)	20 (47%)	8 (30%)	−0.168 [−0.414 to 0.077]	0.175
≥70 [n (%)]	17 (25%)	9 (21%)	8 (30%)	0.093 [−0.124 to 0.311]	0.395
Women [n (%)]	62 (91%)	38 (90%)	24 (92%)	0.018 [−0.125 to 0.162]	0.799
Body Mass Index (kg/m^2^)	22.4 (3.3)	22.8 (3.4)	21.8 (3.2)	−0.941 [−2.622 to 0.741]	0.268
Disease duration (years)	11.57 (11.14)	12.73 (11.42)	9.692 (10.61)	−3.046 [−8.591 to 2.499]	0.277
1–2 [n (%)]	19 (27%)	12 (28%)	7 (26%)	−0.016 [−0.243 to 0.210]	0.885
3–9 [n (%)]	19 (27%)	9 (21%)	10 (38%)	0.170 [−0.053 to 0.393]	0.132
10–19, n (%)	15 (22%)	9 (21%)	6 (23%)	0.016 [−0.193 to 0.226]	0.876
≥20 [n (%)]	15 (22%)	12 (28%)	3 (11%)	−0.170 [−0.376 to 0.035]	0.103
Steinbrocker class [n (%)]				−0.068 [−0.418 to 0.282]	0.700
1	46 (67%)	27 (64%)	19 (73%)	0.088 [−0.148 to 0.324]	0.459
2	16 (23%)	11 (26%)	5 (19%)	−0.070 [−0.283 to 0.144]	0.518
3	5 (7%)	4 (9%)	1 (3%)	−0.057 [−0.188 to 0.074]	0.391
4	1 (1%)	0 (0%)	1 (3%)	0.030 [−0.022 to 0.099]	0.206
Drugs in use [n (%)]					
Non-steroidal anti-inflammatory drugs	38 (55)%	24 (79%)	14 (53%)	−0.033 [−0.284 to 0.218]	0.794
Glucocorticoids	33 (48%)	18 (24%)	15 (57%)	0.148 [−0.102 to 0.398]	0.241
Methotrexate	48 (70%)	27 (15%)	21 (80%)	0.165 [−0.062 to 0.392]	0.152
Biologic agents	20 (29%)	11 (46%)	9 (34%)	0.084 [−0.145 to 0.314]	0.466
Tender joint count (number)	3.54 (3.97)	3.61 (4.45)	3.42 (3.13)	−0.196 [−2.193 to 1.801]	0.845
Swollen joint count (number)	2.38 (3.30)	2.69 (3.87)	1.88 (2.04)	−0.806 [−2.453 to 0.842]	0.332
Patient global assessment (PGA) (mm)	42.55 (26.74)	30.45 (24.07)	62.11 (17.97)	31.663 [20.721 – 42.605]	<0.001*
Physician global assessment (mm)	35.13 (24.93)	39.5 (28.38)	28.07 (16.13)	−11.423 [−23.619 to 0.773]	0.066
C-reactive protein (mg/dL)	1.20 (1.91)	1.41 (2.15)	0.85 (1.42)	−0.562 [−1.513 to 0.388]	0.242
ESR (mm/hour)	30.95 (26.44)	31.80 (27.24)	29.57 (25.57)	−2.233 [−15.496 to 11.031]	0.738
DAS28-ESR (points)	3.97 (1.23)	3.85 (1.28)	4.16 (1.13)	0.306 [−0.307 to 0.919]	0.322
Simplified Disease Activity Index (points)	14.90 (10.09)	14.72 (12.04)	15.18 (5.936)	0.460 [−4.609 to 5.529]	0.857
mHAQ (points)	0.47 (0.59)	0.42 (0.56)	0.56 (0.62)	0.142 [−0.153 to 0.436]	0.341
Nine-year follow-up					
Working [n (%)]	21 (30%)	12 (28%)	9 (34%)	0.060 [−0.173 to 0.294]	0.607
Presence of pain [n (%)]	65 (95%)	39 (16%)	26 (16%)	0.071 [−0.031 to 0.174]	0.168
Knee	38 (55%)	23 (54%)	15 (57%)	0.029 [−0.222 to 0.280]	0.816
Hand	32 (47%)	18 (42%)	14 (38%)	0.110 [−0.141 to 0.361]	0.385
Neck	30 (44%)	18 (42%)	12 (46%)	0.033 [−0.218 to 0.284]	0.794
Back	27 (39%)	15 (35%)	12 (46%)	0.104 [−0.142 to 0.351]	0.400
Foot	25 (36%)	11 (26%)	14 (38%)	0.277 [0.042 – 0.511]	0.021*
Ankle	12 (17%)	8 (19%)	4 (15%)	−0.037 [−0.229 to 0.156]	0.705
Wrist	9 (13%)	6 (14%)	3 (11%)	−0.027 [−0.199 to 0.144]	0.750
Elbow	9 (13%)	5 (11%)	4 (15%)	0.035 [−0.136 to 0.206]	0.686
Shoulder	6 (8%)	5 (11%)	1 (3%)	−0.081 [−0.223 to 0.061]	0.262
Pain-NRS (points)	3.29 (2.19)	2.71 (1.97)	4.23 (2.25)	1.516 [0.477 – 2.556]	0.005*
PCS (points)	19.22 (13.49)	15.69 (12.08)	24.92 (13.91)	9.233 [2.851 – 15.614]	0.005*
Rumination (points)	9.17 (5.78)	7.85 (5.41)	11.30 (5.81)	3.451 [0.675 – 6.226]	0.016*
Helplessness (points)	6.01 (5.27)	4.83 (4.90)	7.92 (5.39)	3.090 [0.551 – 5.628]	0.018*
Magnification (points)	4.02 (3.14)	3 (2.66)	5.69 (3.19)	2.692 [1.257 – 4.127]	<0.001*
HADS (points)	10.38 (6.88)	9.52 (6.22)	11.76 (7.74)	2.245 [−1.164 to 5.655]	0.193
Anxiety (points)	4.60 (3.60)	4.14 (3.20)	5.34 (4.12)	1.203 [−0.582 to 2.988]	0.183
Depression (points)	5.77 (3.98)	5.38 (3.88)	6.42 (4.11)	1.042 [−0.940 to 3.024]	0.298
PDAS (points)	18.01 (14.18)	15.73 (11.63)	21.69 (17.15)	5.954 [−1.013 to 12.921]	0.093
PSEQ (points)	37.73 (12.80)	40.21 (11.67)	33.80 (13.73)	−6.412 [−12.674 to −0.150]	0.045*
EQ-5D-3L (points)	0.73 (0.19)	0.77 (0.16)	0.66 (0.23)	−0.110 [−0.206 to −0.014]	0.025*

DAS, Disease Activity Score; EQ-5D-3L, European Quality of Life 5 Dimensions 3 Level; ESR, erythrocyte sedimentation rate; mHAQ, modified Health Assessment Questionnaire; NRS, Numerical Rating Scale; PCS, Pain Catastrophizing Scale; PDAS, Pain Disability Assessment Scale; PGA, Patient global assessment; PSEQ, pain self-efficacy questionnaire. Data from continuous variables are presented as mean (standard deviation). Data for categorical variables are presented as numbers (%). *Significance level was set at <5%.

**FIGURE 2:**
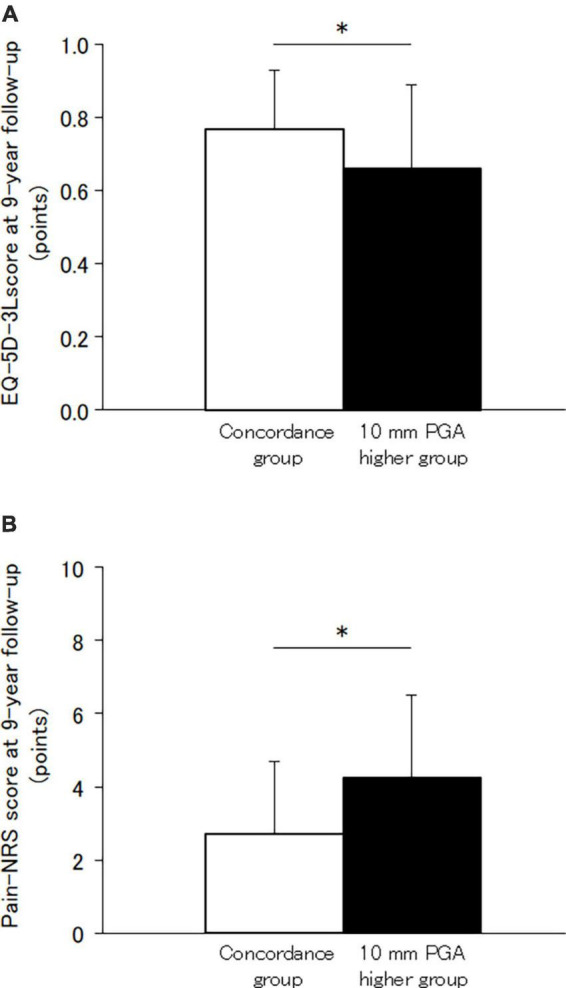
Difference of scores between those with concordance and 10 mm PGA higher than physician global assessment. **(A)** EQ-5D-3L score. **(B)** Pain-NRS values. The EQ-5D-3L score and Pain-NRS values at 9-year follow-up measurement were significantly worse in the 10 mm PGA higher group than the concordance group. *Significance level was set at <5%. EQ-5D-3L, European Quality of Life 5 Dimensions 3 Level; NRS, Numerical Rating Scale; PGA, patient global assessment of disease activity.

At the 9-year follow-up measurement, 65 of the 68 patients (95%) experienced pain. The mean scores were 3.29 for Pain-NRS, and 0.73 for EQ-5D-3L at the 9-year follow-up measurement. The patients with a 10 mm higher PGA than the physician global assessment at baseline measurements had significantly worse pain rating scale, PCS, PSEQ, and EQ-5D-3L scores at the 9-year follow-up measurement compared to those with concordance (Table 1; Figure 2). There were significant differences of 1.5 points and 0.11 points in the NRS values and EQ-5D-3L scale scores between groups, respectively.

The correlations of the EQ-5D-3L scale scores and Pain-NRS scores at the 9-year follow-up with the independent variables at baseline are shown in Table 2. The 10 mm higher PGA and mHAQ values were significantly correlated with the EQ-5D-3L scale scores and Pain-NRS values in the univariable analysis.

**TABLE 2:** Correlations of the EQ-5D-3L scale scores and Pain-NRS value at 9-year follow-up measurement with independent variables at baseline.

	EQ-5D-3L at 9-year follow-up		Pain-NRS at 9-year follow-up	
	Correlation coefficient	*p*-value	Correlation coefficient	*p*-value
Baseline				
Age	−0.121	0.326	0.045	0.716
Women	0.049	0.691	0.137	0.266
Body Mass Index	−0.038	0.759	0.097	0.433
Disease duration	−0.207	0.090	0.103	0.402
Steinbrocker class	−0.114	0.356	0.121	0.325
Non-steroidal anti-inflammatory drugs	−0.004	0.972	−0.057	0.646
Glucocorticoids	−0.158	0.199	0.152	0.215
Methotrexate	0.099	0.422	0.013	0.916
Biologic agents	−0.219	0.073	0.209	0.088
Tender joint count	−0.167	0.173	0.162	0.186
Swollen joint count	−0.190	0.120	0.165	0.179
Patient global assessment (PGA)	−0.186	0.129	0.182	0.137
Physician global assessment	−0.044	0.723	−0.012	0.922
10 mm PGA higher	−0.271	0.025*	0.338	0.005*
C-reactive protein	−0.200	0.103	0.189	0.122
ESR	−0.035	0.776	−0.056	0.647
DAS28-ESR	−0.161	0.189	0.145	0.238
Simplified Disease Activity Index	−0.226	0.064	0.199	0.104
mHAQ	−0.492	<0.001*	0.368	0.002*

DAS, Disease Activity Score; EQ-5D-3L, Euro Quality of life-5 Dimensions-3 level; ESR, erythrocyte sedimentation rate; mHAQ, modified Health Assessment Questionnaire; NRS, Numerical Rating Scale; PGA, Patient global assessment. Data were analyzed using Pearson’s correlation coefficient test. Categorical variables included dummy variables. The 10 mm PGA higher and mHAQ values were significantly correlated with EQ-5D-3L scale scores and Pain-NRS in the univariate analysis. *Significance level was set at <5%.

The results of the multivariate analysis for the EQ-5D-3L scale scores and Pain-NRS values are shown in Table 3. The mHAQ score and 10 mm higher PGA at baseline were significantly independently associated with the EQ-5D-3L scale score and Pain-NRS score at the 9-year follow-up in the multivariable analysis. No multicollinearity was observed for any of the tested independent variables.

**TABLE 3:** Multivariable analysis.

Independent variables	*B*	SE	Beta	*p*-value	*R* ^2^
A) Analysis for EQ-5D-3L scale at 9-year follow-up measurement					
mHAQ	−0.159	0.033	−0.497	<0.001*	0.308
10 mm PGA higher	−0.087	0.042	−0.212	0.043*	
Biologic agents				0.714	
Simplified Disease Activity Index				0.822	
B) Analysis for Pain-NRS at 9-year follow-up measurement					
mHAQ	1.232	0.409	0.328	0.004*	0.230
10 mm PGA higher	1.411	0.488	0.314	0.005*	
Biologic agents				0.588	

B, non-standard regression coefficient; Beta, standardized regression coefficient; EQ-5D-3L, Euro Quality of life-5 Dimensions-3 level; mHAQ, modified Health Assessment Questionnaire; NRS, Numerical Rating Scale; PGA, Patient global assessment; R^2^, multiple correlation coefficient adjusted for degrees of freedom; SE, standard error. These data were analyzed using multivariate analysis. The multicollinearity of the variables was also assessed (correlation coefficient < 0.9). The higher mHAQ score and 10 mm PGA higher at baseline were significantly independently associated with the pain NRS and EQ-5D-3L scale scores at the 9-year follow-up in the multivariable analysis. *Significance level was set at <5%.

## 4. Discussion

### 4.1. Overview

The present longitudinal cohort study suggested that discordance in the global assessment between patients and physicians modestly predicted worse 9-year pain-related outcomes in patients with RA. These findings suggest that the presence of discordance between patients and physicians could predict the treatment outcome of patients with RA.

### 4.2. Discordance between patients and physicians

Many patients are unable to express their disease burdens and treatment goals (32). The patient global assessment is usually worse than the physician global assessment (17). There was moderate discordance between the observed functional disability and self-report questionnaires in patients with RA (33). Increased pain leads to a discrepancy toward worse patient global assessment, while an increased number of swollen and tender joints lead to a discrepancy toward worse physician global assessment (34). High pain, general health, and C-reactive protein levels before treatment are associated with discordance in assessment between patients and physicians after treatment (35). The number of patients with discordance has either not resolved or has increased over time (18, 35). Physician global assessment is often decreased during treatment, whereas patient global assessment is sometimes unchanged (18). The discordance between the patients and physicians is hypothesized to result in patient dissatisfaction, difficulties regarding treatment decision-making, poor adherence, and worse treatment outcomes (17, 34, 35). The patients with discordance had significantly worse pain and pain-related psychological factors at the 9-year follow-up measurement in the present study. A combination of subjective and objective clinical measurements is useful for patients with RA (1). This information may help in the treatment and prognosis of patients with RA.

### 4.3. Physician-patient communication

Physician-patient communication is associated with accurate care as well as with more satisfied patients (36). The clinical expectations for analgesia between patients and physicians are in agreement, with some discordance (37). Most physician-patient communication focuses on symptoms and treatment options rather than the patients’ perspective of quality of life (32). RA remissions of tenderness, swelling, and pain are consistently associated with physician assessment but not patient-reported outcomes (38). Physicians should initiate more detailed discussions with patients regarding expectations and carefully explain treatment-to-target approaches and other goal-setting strategies (32). Specifically, patients with inadequate health literacy are likely to report poor communication in the domains of general clarity, explanation of their condition, and processes of care (39). Physician-patient communication is expected to be a shared control in patients with adequate health literacy; however, physician dominance and patient passivity sometimes occur in patients with inadequate health literacy (40). The sex of the patient and physician could impact the physician-patient interaction and its outcomes (41). Discordance was more common in female patients, regardless of the sex or age of the physician (42). Many physicians tend to overestimate their communication (36). Physicians with better communication and interpersonal skills can detect problems earlier, prevent medical crises and expensive interventions, and provide better support to their patients (36). Furthermore, wearable activity trackers provide objective data for healthcare providers and for patients to educate themselves (43). The objective measurements of physical activity and sleep might resolve the discordance between patients and physicians. Encouraging and educating patients may play a key role in improving psychological disturbance and emotional wellbeing (44). The evaluation of a bio-psychosocial framework enhances the evaluation of the health-related quality of life and disability in the clinical management of patients (8).

### 4.4. Variety of discordance between patients and physicians

The impact of discordance between patients and physicians on treatment outcomes has been shown in Asian patients with early RA (18), and is further established for RA in the present study. Ethics and sociocultural contexts are associated with pain and health perceptions in patients (45). The discordance between patient and physician ratings varies widely across different countries (17). The degree of discordance in the global assessment was relatively small in the present study, which is consistent with the results of previous studies (18). Patients with osteoarthritis are more likely to be discordant (46), similar to those with RA (47). Physicians mainly assess the patient’s experience of pain and other symptoms of osteoarthritis because laboratory findings are not informative for the diagnosis and management of osteoarthritis (46). The effect of discordance between physicians and patients on treatment outcomes has not been demonstrated across different diseases, countries, and cultures.

### 4.5. Study limitations

The present study has several limitations. First, the courses of the global assessment of disease by patients and physicians, disease activity, physical and psychological disturbances, and objective measurements were not investigated. Second, the present study excluded the participants who were not returning or not completing the follow-up measurement by mail. Third, the follow-up measurements were by mail and not face-to-face, which had a response bias. Fourth, the present study included patients with established RA at different stages of disease activity. Finally, this study included only a small number of patients. Therefore, the observations should be interpreted with caution.

## 5. Conclusion

In conclusion, discordance in global assessment between patients and physicians modestly predicted worse 9-year pain-related outcomes in patients with RA. This finding suggests the importance of discordance in global assessment between patients and physicians in patients with RA.

## Data availability statement

The raw data supporting the conclusions of this article will be made available by the authors, without undue reservation.

## Ethics statement

The studies involving human participants were reviewed and approved by the Research Ethics Committee of Hayaishi Hospital. Written informed consent for participation was not required for this study in accordance with the national legislation and the institutional requirements.

## Author contributions

KH and KM designed the study and wrote the main manuscript. KS, MY, MH, and YH prepared and supervised the analyses. All authors meet the International Committee of Medical Journal Editors (ICMJE) criteria for authorship of this article, take responsibility for the integrity of the work as a whole, and have approved this version to be published.
